# *In vivo* Protein Corona Formation: Characterizations, Effects on Engineered Nanoparticles’ Biobehaviors, and Applications

**DOI:** 10.3389/fbioe.2021.646708

**Published:** 2021-03-31

**Authors:** Xue Bai, Jiali Wang, Qingxin Mu, Gaoxing Su

**Affiliations:** ^1^School of Public Health, Cheeloo College of Medicine, Shandong University, Jinan, China; ^2^School of Pharmacy, Nantong University, Nantong, China; ^3^Department of Pharmaceutics, University of Washington, Seattle, WA, United States

**Keywords:** protein corona, engineered nanoparticles, nano-bio interactions, nanomedicine, biobehaviors, nanotoxicology

## Abstract

Understanding the basic interactions between engineered nanoparticles (ENPs) and biological systems is essential for evaluating ENPs’ safety and developing better nanomedicine. Profound interactions between ENPs and biomolecules such as proteins are inevitable to occur when ENPs are administered or exposed to biological systems, for example, through intravenous injection, oral, or respiration. As a key component of these interactions, protein corona (PC) is immediately formed surrounding the outlayer of ENPs. PC formation is crucial because it gives ENPs a new biological identity by altering not only the physiochemical properties, but also the biobehaviors of ENPs. In the past two decades, most investigations about PC formation were carried out with *in vitro* systems which could not represent the true events occurring within *in vivo* systems. Most recently, studies of *in vivo* PC formation were reported, and it was found that the protein compositions and structures were very different from those formed *in vitro*. Herein, we provide an in-time review of the recent investigations of this *in vivo* PC formation of ENPs. In this review, commonly used characterization methods and compositions of *in vivo* PC are summarized firstly. Next, we highlight the impacts of the *in vivo* PC formation on absorption, blood circulation, biodistribution, metabolism, and toxicity of administered ENPs. We also introduce the applications of modulating *in vivo* PC formation in nanomedicine. We further discuss the challenges and future perspectives.

## Introduction

Engineered nanoparticles (ENPs) with unique physical and chemical properties have been widely developed in the fields of energy (Pomerantseva et al., 2019; Wang et al., 2020), electronics (Kamyshny and Magdassi, 2019), materials (Wang et al., 2019), biomedicine (Lee et al., 2012; Pelaz et al., 2017; Wong et al., 2020), food and agriculture (Peters et al., 2016; Lowry et al., 2019), and so on. More and more ENP-based biomedical products were put into the markets or translated to clinic. Despite the rapid developments in these fields, there remain a number of challenges in nanotechnology development. The key issues are the health risk and safety concern of ENPs, especially in the field of nanomedicine (Susan et al., 2018; Ahmad et al., 2019). Numerous studies have reported the ENP-associated toxicity to zebra fish (Lakshmi et al., 2019; Bakshi, 2020), macroalgae (Sfriso et al., 2019), animals (Jia et al., 2017; Teng et al., 2019), and even human beings (Guo et al., 2020). To avoid these ENP-caused toxicities and promote the development of nanomedicine, it is imperative to thoroughly understand the basic interactions between ENPs and the physiological environment, biomolecules, tissues, and organs. Therefore, investigations about ENPs-physiological systems interactions stay at the forefront of nanomedicine and nanotoxicology research.

Once entering into the physiological environment, including blood, interstitial fluid, and intracellular environment, ENPs are expected to interact with biomolecules such as lipids, metabolites, sugars, and especially proteins by van der Waals forces, electrostatic, hydrogen bonding, and hydrophilic/hydrophobic interactions because of the high surface energy and unique surface chemistry of ENPs, and after that biomolecular corona are formed (Nel et al., 2009; Monopoli et al., 2012; Shemetov et al., 2012; Hadjidemetriou and Kostarelos, 2017). Until now, the protein corona (PC) is the most important class of biomolecular corona formed and has mostly been studied. Before the PC was proposed in 2007, ENPs had been considered to interact with living cells directly, and the physiochemical properties of the pristine ENPs were considered to determine their biological effects (Cedervall et al., 2007). However, this understanding is far from the truth. After exposed to a complete cell culture medium *in vitro* or blood *in vivo*, tens of thousands of proteins immediately reached onto the outlayer of ENPs and PC were formed in a dynamic process (Yu et al., 2020). According to the relative affinity, PC was divided into hard and soft types (Docter et al., 2015; Kokkinopoulou et al., 2017). Hard corona is made by a protein fraction strongly bound to the surface, while soft corona is formed by loosely bound proteins, maybe via protein–protein interaction. PC significantly alters the identity of ENPs, including physicochemical properties and the bioidentity of ENPs (Caracciolo G. et al., 2016; Liu et al., 2020; Szekeres et al., 2020). First, PC formation alters the size, zeta potential, morphology, and aggregation states of ENPs. Second, PC formation alters the interactions between ENPs and physiological systems, and the subsequent biobehaviors of ENPs (Chinen et al., 2017; Nayak et al., 2019). For example, adsorption of opsonin proteins enhanced the cellular uptake of ENPs by phagocytes, and subsequently accelerate the clearance (Vu et al., 2019). In addition, special protein can be recruited by ENPs with special properties which give ENPs specificity capacity (Wang et al., 2017). Therefore, investigation of interaction mechanism between ENPs and protein molecules and its biological effects is critical to understand the interactions between ENPs and living organisms, which is of great significance for evaluating the safety of ENPs and the application of ENPs in biomedical field.

Until now, most of studies are investigated on the PC formation on ENPs and their biological effects *in vitro*. However, there are significant differences between *in vitro* and *in vivo* PC formation, not only quantities of adsorbed protein, but also the composition and structures (Amici et al., 2017; Wang M. et al., 2018). Furthermore, the subsequent impacts on bio-nano interactions are also vary *in vitro* and *in vivo* (Raoufi et al., 2018). Thus, in this review, we focus on the research of *in vivo* PC formation and their impacts on the biobehaviors of administered ENPs. To investigate the *in vivo* PC formation, characterization techniques are different from the *in vitro* PC analytical approaches, which were discussed in the first part. Next, we summarize the influences of *in vivo* PC formation on absorption, blood circulation, biodistribution, metabolism, and toxicity of administered or exposed ENPs. Moreover, *in vivo* PC formation can be modulated by the physicochemical properties of ENPs. We then further discuss the applications of *in vivo* PC formation for targeted delivery and personalized medicine. Finally, the major research gaps, challenges, and future perspectives of *in vivo* PC formation are discussed.

## Characterization of *in vivo* PC Formation

Characterization of *in vivo* PC formation and protein-bound ENPs is a key step to understand the formation mechanism and the function of protein composition (Brun and Sicard-Roselli, 2014; Kokkinopoulou et al., 2017). Analytical methods of PC protein composition and structure can be categorized into *in situ* and *ex situ* characterizations (Sakulkhu et al., 2014; Carril et al., 2017). *Ex situ* techniques separate protein-bound ENPs from the physiological environment and then cleave the bound proteins for further characterization. On the contrary, *in situ* technique directly provide relevant information about PC formation when ENPs disperse into physiological environment.

For *ex situ* characterization, separation of PC-ENP complexes from *in vivo* physiology environment is one of the main obstacles in characterizing the *in vivo* PC formation. Magnetic separation method was employed to separate protein-bound magnetic ENPs, which can avoid disrupting loosely bound protein during centrifugation. Sakulkhu et al. (2014) employed magnetic separation to obtain PC-ENP complexes from rat sera, and then PC compositions were analyzed by nano-LC-MS/MS. They found that ENPs with positive charge adsorbed 32 types of proteins, while neutral and negative charged ENPs adsorbed 55 and 51 types of proteins, respectively. Low molecular weight (<30 kDa) proteins are the most amount of proteins for all ENPs adsorbed *in vivo*, while 90–120 kDa proteins were the least. The top 5 bound proteins were listed in Table 1. Hemoglobin subunit, such as alpha-1/2, beta-1, and beta-2 are all in the top 5 bound proteins. Size-exclusion chromatography (SEC) combined with membrane ultrafiltration were usually employed to separate non-magnetic protein-bound ENPs. For example, PC-AuNP complexes recovered from blood can be separated from unbound proteins by SEC, followed by membrane ultrafiltration (García-Álvarez et al., 2018). They found that the total number of identified proteins were 298 and 246 for AuNRs with diameters of 40 and 70 nm, respectively. AuNSs with diameters of 40 nm and 70 nm adsorbed 406 and 215 types of proteins, respectively. Serum albumin and alpha-2-macroglobulin were the top 2 bound proteins for all AuNPs (Table 1). Proteins with MW < 80 kDa contributed to 75–80% of the PC. Besides, PC-liposome complexes can also be separated form plasma by SEC. The types of unique proteins identified *in vivo* formed coronas of bare-, PEG-, and monoclonal antibody targeted-liposomes NPs were 453, 478, and 511, respectively (Hadjidemetriou et al., 2015). Apolipoproteins were the most abundant classes of protein *in vivo* PC of all types of liposomes NPs. Separation of protein-bound ENPs from plasma may inevitably interfere with the composition of the PC, resulting in the loss of weak-binding proteins, which is inaccurate for the subsequent analysis. It is also difficult to separate PC-ENP complexes from tissues/organs other than blood.

**TABLE 1:** Top-five adsorbed proteins of some ENPs with different properties and the separation methods.

**ENPs**	**Properties**	**Administration route and sampling time**	**Separation method**	**Top-five adsorbed proteins (Relative protein abundance)**	**References**
SPIONs	90 nm, positive charged	10 min after intravenous injection	magnetic separation	Hemoglobin subunit beta-2 (16.76%) Hemoglobin subunit alpha-1/2 (16.50%) Hemoglobin subunit beta-1 (13.04%) Apolipoprotein E (9.99%) Fibrinogen alpha chain (7.90)	Sakulkhu et al., 2014
	95 nm, neutral	10 min after intravenous injection	magnetic separation	Fibrinogen beta chain (9.05%) Hemoglobin subunit beta-2 (8.86%) Hemoglobin subunit alpha-1/2 (8.44%) Fibrinogen gamma chain (7.30%) Hemoglobin subunit beta-1 (6.94%)	
	91 nm, negative charged	10 min after intravenous injection	magnetic separation	Hemoglobin subunit beta-2 (10.23%) Hemoglobin subunit alpha-1/2 (9.33%) Hemoglobin subunit beta-1 (8.96%) Apolipoprotein A-II (7.15%) Apolipoprotein E (5.72%)	
AuNRs	40 nm, PEG-COOH coated	10 min after intravenous injection	SEC and membrane ultrafiltration	Serum albumin (5.16%) Alpha-2-macroglobulin (4.30%) Fibrinogen beta chain (2.29%) Apolipoprotein A-I (2.27%) Complement factor H (2.12%)	García-Álvarez et al., 2018
	70 nm, PEG-COOH coated	10 min after intravenous injection	SEC and membrane ultrafiltration	Serum albumin (7.52%) Alpha-2-macroglobulin (6.13%) Serine protease inhibitor A3K (4.83%) Apolipoprotein A-I (3.24%) Fibrinogen beta chain (2.51%)	
AuNSs	40 nm, PEG-COOH coated	10 min after intravenous injection	SEC and membrane ultrafiltration	Serum albumin (3.71%) Alpha-2-macroglobulin (3.70%) Serine protease inhibitor A3K (2.44%) Fibrinogen beta chain (2.37%) Apolipoprotein E (2.31%)	
	70 nm, PEG-COOH coated	10 min after intravenous injection	SEC and membrane ultrafiltration	Serum albumin (8.19%) Alpha-2-macroglobulin (7.80%) Serine protease inhibitor A3K (6.01%) Fibrinogen beta chain (3.99%) Alpha-1B-glycoprotein (3.11%)	
AmBisome^<^	100 nm	10 min after intravenous injection	SEC and membrane ultrafiltration	Serum albumin (4.07%) Fibrinogen beta chain (2.28%) Apolipoprotein C-III (2.14%) Actin, cytoplasmic 2 (2.11%) Fibrinogen gamma chain (1.81%)	Amici et al., 2017
polystyrene NPs	80.77 nm, PEG coated	10 min after intravenous injection	SEC and membrane ultrafiltration	ApoE protein (9.83%) Apolipoprotein C-IV (7.56%) Apolipoprotein A-IV (7.29%) Clusterin (5.31%) Hemoglobin subunit beta-2 (4.08%)	Zhang et al., 2018
	89.5 nm, modified with LT7	10 min after intravenous injection	SEC and membrane ultrafiltration	ApoE protein (7.54%) Apolipoprotein A-IV (7.54%) Clusterin (6.62%) Albumin 1 (3.89%) Apolipoprotein C-IV (2.59%)	
	83.61 nm Modified with DT7	10 min after intravenous injection	SEC and membrane ultrafiltration	Apolipoprotein A-IV (7.14%) Clusterin (6.57%) ApoE protein (6.32%) Apolipoprotein C-IV (2.52%) Albumin 1 (2.34%)	
	92.47 nm, modified with Tf	10 min after intravenous injection	SEC and membrane ultrafiltration	Hemoglobin subunit beta-2 (8.65%) Albumin 1 (7.69%) Clusterin (5.22%) Apolipoprotein A-IV (5.20%) ApoE protein (4.61%)	
Liposome	127.77 nm	10 min after intravenous injection	SEC and membrane ultrafiltration	Apolipoprotein C-III (4.93%) Apolipoprotein E (3.54%) Hemoglobin subunit beta-1 (3.34%) Beta-globin (3.17%) Alpha-2-macroglobulin (2.96%)	Hadjidemetriou et al., 2015
	119.53 nm, PEG coated,	10 min after intravenous injection	SEC and membrane ultrafiltration	Apolipoprotein C-III (4.53%) Apolipoprotein E (3.46%) Hemoglobin subunit beta-1 (2.89%) Alpha-globin 1 (2.24%) Alpha-2-macroglobulin (2.15%)	
	121.73 nm, monoclonal antibody (IgG) targeted	10 min after intravenous injection	SEC and membrane ultrafiltration	Apolipoprotein E (2.79%) Apolipoprotein C-III (2.66%) Alpha-2-macroglobulin (2.49%) Hemoglobin subunit beta-1 (2.44%) Apolipoprotein C-IV (1.58%)	
PEGylated liposomal doxorubicin	115 nm	10 min after intravenous injection	SEC and membrane ultrafiltration	Alpha-2-macroglobulin (8.02%) Apolipoprotein C-III (6.37%) Hemoglobin subunit beta-1 (5.79%) Apolipoprotein E (PE = 1 SV = 2) (5.57%) Beta-globin, Hbbt1 (A8DUK2) (4.48%)	Hadjidemetriou et al., 2016
	115 nm	1 h after intravenous injection	SEC and membrane ultrafiltration	Apolipoprotein E (PE = 2 SV = 1) (8.19%) Alpha-2-macroglobulin (7.66%) Apolipoprotein C-III (4.86%) Serum albumin (4.41%) Apolipoprotein E (PE = 1 SV = 2) (3.87%)	
	115 nm	3 h after intravenous injection	SEC and membrane ultrafiltration	Hemoglobin subunit beta-1 (8.58%) Apolipoprotein E (PE = 2 SV = 1) (7.30%) Apolipoprotein C-III (6.65%) Alpha-2-macroglobulin (6.42%) Beta-globin, Hbbt1 (A8DUK2) (6.02%)	

After separation, the characterization methods for protein-bound ENPs and cleaved proteins were similar to that used in *in vitro* PC analysis. For example, size of PC-ENP complexes can be characterized by transmission electron microscopy (TEM) (Mahmoudi et al., 2011) and atomic force microscopy (AFM) (Guan et al., 2015). Polyacrylamide gel electrophoresis (PAGE) and liquid chromatography tandem mass spectrometry (LC-MS/MS) (Walkey et al., 2014; Saha et al., 2016; Pinals et al., 2020) are commonly used for the identification and quantification of individual proteins in the PC after the separation of adsorbed proteins from the surface of ENPs. Isothermal titration calorimetry (ITC) (Fleischer and Payne, 2014) and SEC (Shakiba et al., 2018) can be used to evaluate the strength and adsorption kinetics of the interaction between ENPs and proteins. Furthermore, conformational changes of proteins was determined by circular dichroism (CD) spectroscopy (Yan et al., 2013; Fleischer and Payne, 2014) and fourier transform infrared spectroscopy (FTIR) (Wang et al., 2012), nuclear magnetic resonance (NRM) (Brancolini et al., 2012), and enzyme activity determination (Gagner et al., 2011). These traditional techniques are used to determine the structural and physicochemical parameters of PC.

Unlike *ex situ* technique, *in situ* technique may be more suitable for characterization of PC in biofluids. For example, the current understanding of the biological identity that ENPs may acquire in a given biological milieu is mostly inferred from hard corona. However, because classical approach based on ENPs separation from the biological medium fails to detect the composition of soft corona and illustrate their biological relevance. In recent, *in situ* techniques were employed to character soft corona. For example, a new method using cryoTEM and synchrotron-radiation CD was developed to analyze the weakly bound proteins and reveal molecular basis of soft corona *in situ* (Sanchez-Guzman et al., 2020). Soft corona proteins were altered by ENPs based on shifting the equilibrium toward the unfolded states at physiological temperature. Besides, *in situ* click-chemistry reaction was also used to identify soft corona and hard corona on the surface of silica and polystyrene NPs (Mohammad-Beigi et al., 2020). They found that soft corona and hard corona were distinguished by different binding strength but not special proteins.

In addition, the hydrodynamic radius of PC-ENP complexes in biofluid can be analyzed by ^19^F diffusion-ordered NMR (Carril et al., 2017), fluorescence correlation spectroscopy (Shang and Nienhaus, 2017) and high-speed dark-field microscopy (Lin et al., 2019). Molecular motifs can be detected by flow cytometry combined with microfluidics in biological milieu (Lo Giudice et al., 2016). Protein adsorption behavior, including affinity and adsorption orientation can be analyzed by *in situ* fluorescence resonance energy transfer (FRET) (Qu et al., 2020). Time-evolution of PC formation can be detected by microfluidics combined with confocal laser scanning microscopy (Weiss et al., 2019).

Despite these technologies are established to analyze the ENP-bound PC formation, several limitations remain. First, most of them don’t take the protein conformational changes into account. In fact, the formation of PC-ENP complexes can affect the higher structures of protein molecules. For example, secondary structures of bovine serum albumin (BSA) molecules were broken by Au NPs and Ag NPs after adsorbing on NPs (Treuel et al., 2010). Carbon nanotubes induced significant changes in the secondary structure of bovine fibrinogen and Ig, with a decrease in the α-helical content and an increase in the β-sheet (Ge et al., 2011). Therefore, the *in vivo* conformational changes of PC should be considered in the analysis process. Second, the analysis of PC formation is still lacking a proper modeling of the interaction kinetics. This is a challenging task because a large number of molecules are involved and dynamic exchanges occur in a short period of time. Only scarce studies are carried out in this regard. For example, the kinetics of the PC formed on Silica NPs were studied by combing experiments with simulations and theory in a ternary solution made of HSA, Tf, and Fib. Theoretical model correctly reproduced competitive protein replacement and observed a memory effect in the final corona composition, which was proved by independent experiments (Vilanova et al., 2016). Besides, Duan et al. (2020) found a descriptor based on fluorescence change from fluorescamine labeling on a protein, which helped build machine learning models to predict the composition of PC, even in heterogeneous nanomaterials. More appropriate models need to be investigated to predict PC behaviors and their influence on nano-bio interactions in complex environment *in vivo*.

In our opinion, obtaining an accurate PC information *in vivo* remains a challenge due to the rapid and dynamic, complex and often weak interactions between ENPs and proteins. Development of appropriate *in situ* analytical approaches that can address these issues are highly demanded.

## Impacts of *in vivo* PC on Biobehaviors of Administered ENPs

### Absorption

Absorption is the process in which ENPs enters the blood from the site of administration. Intravenous injection (IV) seems to be the most recurrent application route for most ENPs and this process is not related to the absorption. However, PC formation should be taken into account in non-IV routes of administration such as oral and inhale. After non-IV routes of administration, ENPs must translocate through the mucus layer before reaching the surface of the epithelium, and then transepithelial absorption may occur (García-Díaz et al., 2017). So far, most of reports focused on the study of PC upon plasma or blood contact, whereas studies involving the biomolecular corona in non-IV administration, especially studies *in vivo* are still scarce.

**FIGURE 1:**
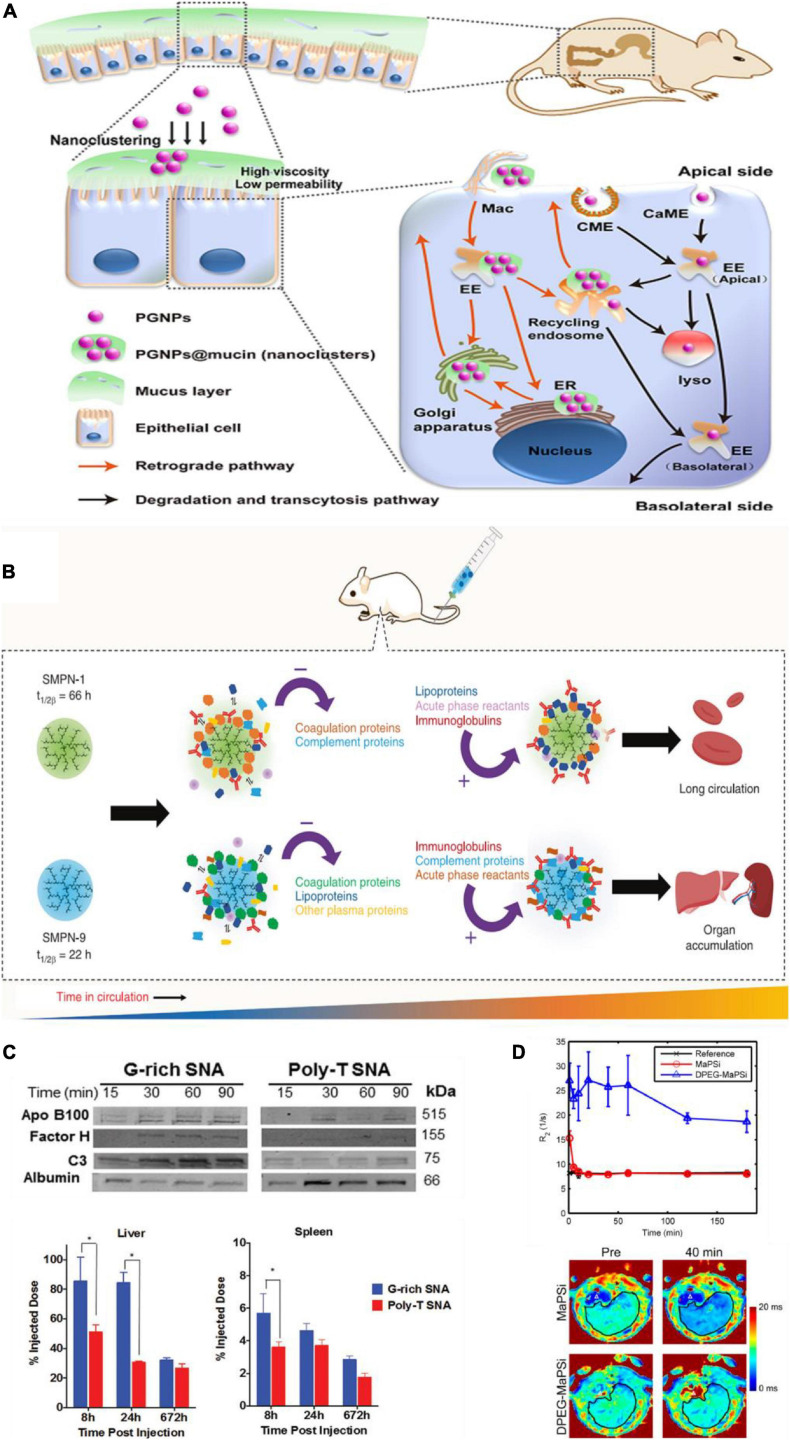
Protein corona impacts *in vivo* biobehaviors of ENPs. **(A)** Schematic diagram of intestinal mucus altering the endocytosis and transcytosis characteristics of PGNPs. Adapted with permission from Yang et al. (2018). **(B)** PC affects circulation time. Coagulation and complement proteins that were adsorbed initially on the surface of SMPN-1 were released during the circulation and accumulation of lipoproteins and acute phase reactants happened over 48 h on the surface. In contrast, short-circulating SMPN-9 had high abundance of complement proteins and acute phase reactants at 48 h. Adapted with permission from Abbina et al. (2020). **(C)** PC influenced the biodistribution of G-rich and poly-T SNAs. Compared to poly-T SNAs, G-rich SNAs adsorbed more amounts of apolipoprotein B100, complement factor H, and complement C3, and less human serum albumin, which induced more accumulation in liver and spleen. *p < 0.05. Adapted with permission from Chinen et al. (2017). **(D)** The major part of MaPSi was cleared fast from liver just a few minutes after injection, whereas DPEG-MaPSi decreased the removal rate and prevented the fast decline of theT_2_* relaxation rates. Adapted with permission from Nissinen et al. (2016).

After oral administration, ENPs encounter gastrointestinal (GI) fluids with multiple components including zwitterionic phospholipids, bile salts, carbohydrates, and mucin. These GI fluids can adsorb on the surface of ENPs, and therefore, impact their properties and may impede their passage through the intestinal mucosa. For example, after oral administration of PEG-GNPs (PGNPs), intestinal mucin adsorbed on their surface and induced aggregation to form nanoclustering (Figure 1A; Yang et al., 2018). Compared with the monodispersive PGNPs, mucin adsorbed nanoclustering entered into epithelial cell through endocytosis, and transported through retrograde pathway [PM-Golgi/ER-PM (plasma membrane)]. Therefore, transcellular capability of PGNP was inhibited by mucin, while cell uptake by epithelial cell was enhanced. Covered ENPs with native proteins could prevent the adsorption of mucus. Wang A. et al. (2018) found that pre-coating of liposomes with a corona of BSA could improve their mucus-penetration and intestinal absorption ability.

The respiratory tract lining fluid is the first biological matter that ENPs encounter after inhale route of administration. This layer is mainly composed of pulmonary surfactants which is a complex mixture of phospholipids, proteins, and cholesterol. The adsorption of these components on inhaled ENPs likely modulates the bioavailability. One study found that polyethylene glycol-coated AuNPs with different outermost regions (Au@PEG-X NPs) adsorb similar types of bronchoalveolar lavage fluid (BALF) proteins, but different composition of PC (Yin et al., 2019). PC composition such as surfactant protein D (SP-D), carbonyl reductase (NADPH), albumin and surfactant protein A (SP-A), have strong correlations with intrapulmonary cellular uptake. For example, adsorption of SP-D may promote the cell uptake by neutrophils in the lavaged lung. Adsorption of NADPH may promote the cell uptake by total macrophage and M2 macrophage in the lavaged lung, but have the opposite effect for macrophage in BALF. At the same time, albumin in the PC shows strong negative correlations with the association of Au@PEG-X NPs to dendritic cells and endothelial cell in the lavaged lung, as well as neutrophils in BALF. SP-A in the PC shows strong positive correlation with the association of Au@PEG-X NPs to dendritic cells in BALF. However, another study reported that SP-A had no effect on cell uptake of PLGA/PLA@PEG NPs by adherent cells in BALF (Ruge et al., 2016).

These studies indicate that the absorbed mucus components can affect epithelial uptake of the ENPs and alter kinetics of the ENPs’ transportation. Thus, it is critical to understand whether the process of PC formation during ENP absorption would subsequently modulates the behavior and fate of ENPs *in vivo*, such as blood circulation, biodistribution and metabolism. In many cases, the *in vivo* performance of ENPs does not meet expectations although passed *in vitro* examinations because of overlooking this issue. Therefore, the transport through the mucus layer may be the first factor to consider for design of ENPs in non-IV administered nanomedicine.

### Blood Circulation

The ENPs enter blood following absorption. The formation of PC might change the interactions between ENPs and tissues/cells (Walkey et al., 2014). Thus, PC will consequently impact the circulation time of ENPs in the body. Compositions of PC such as complement protein, fibrinogen and immune globulin, which called opsonin, may enhance the eliminated of ENPs through the reticuloendothelial system (RES) (Vu et al., 2019). If the amount of these proteins in PC is high, it will accelerate the identification and phagocytosis of ENPs by macrophages, resulting in quick clearing from the body. On the contrary, if the PC is rich in another type of protein such as albumin and apolipoprotein, the blood circulation time of NPs will be prolonged *in vivo* (Bertrand et al., 2017).

PEG and other polymer modifications on ENPs are widely used to reduce protein adsorption, complement activation, and therefore prolong ENP circulation time in blood. For example, Zhou et al. (2018) presented a hierarchical PEG modified ENPs which significantly enhance ENPs circulation time compared to ENPs with signal PEG layer. The fluctuating PEG segments reduce protein binding affinity with ENPs, rather than the total amount of adsorbed proteins, which could prolong circulation time. Except for PEG, soft single molecule polymer nanomaterials (SMPNs) which were prepared with macroinitiator, hyperbranched polyglycerol (HPG), was also presented to enhance circulation time in blood (Figure 1B; Abbina et al., 2020). PC composition of ENPs at the biointerface is highly dynamic and remodeled while in circulation. SMPNs with longer circulation are able to clear some of the opsonins that adsorbed on the surface of ENPs initially, which then evade the immune system and can prolong circulation time in blood.

In addition, biomimetic liposomes and leukosomes (which was obtained from leukocyte membrane), were also used to prolong blood circulation time (Corbo et al., 2017). The amount and number of protein types adsorbed on liposomes were about 45 and 42% more than that of leukosomes respectively 1 h after injections. Although compositions of PC, such as immunoglobulin gamma (IgG), coagulation factors, and complement proteins have been identified on both liposomes and leukosomes, blood circulation time of leukosomes was longer than that of liposomes. One reason was the different abundance of these proteins in the PCs of leukosomes and liposomes. Another reason was that IgG and other proteins were adsorbed on leukosomes through a manner of receptor-to-ligand binding, which reduced the interactions with macrophages and avoid opsonization process. Generally, construction of NPs for medicine applications should consider reducing or controlling adsorption of total proteins, especially opsonins, or pre-coating NPs with selected proteins, so as to prolong the blood circulation time, which is a key factor to improve the performance of NPs *in vivo*.

### Biodistribution

Biodistribution is the process in which protein-absorbed ENPs circulating from blood to tissue fluids or intracellular fluids. Generally, based on the process of opsonization, the main biodistribution organs of ENPs are liver and spleen. For example, guaninerich (G-rich) oligonucleotides and poly-thymine (poly-T) oligonucleotides were used to modify AuNPs, and two spherical nucleic acids (SNAs) were obtained, named G-rich SNAs and poly-T SNAs. It was found that G-rich SNAs adsorbed more proteins than poly-T SNAs both in amounts and in types, including complement proteins, which caused more NPs accumulate in the liver and spleen (Figure 1C; Chinen et al., 2017). Eight hour after injection, more than 85% of the injected dose of G-rich SNAs was found in the liver and spleen, compared to approximately 55% of the injected dose of poly-T SNAs. Moreover, in IgM sensitized mice, anti-PEG IgMs coated ENPs adsorbed complement proteins, such as C1q-A, C1q-B, and C1q-C, which involved in classical complement activation pathway, and the amounts were 1.3-fold more than those in control IgM naive mice. This difference caused the amount of PEGylated NPs accumulated in liver in IgM sensitized mice to increase nearly 3 folds compared with that in control mice (Grenier et al., 2018).

On the contrary, conjugation with blood proteins, such as albumin and apolipoprotein E, remarkably reduces recognition of ENPs by mononuclear phagocytic system. One study found that liver retention of polyelectrolyte-multilayer-coated AuNPs with a diameter of 15 nm was reduced by about 23 and 43% after NPs were conjugated with apolipoprotein E and albumin (Schäffler et al., 2014). Furthermore, albumin conjugation significantly increases translocation into the brain and lung. However, another study found that despite pre-incubated with mouse serum before administration, CTAB-AuNRs mainly accumulated in liver (Cai et al., 2016). Some of the CTAB-AuNRs escaped the phagocytosis mainly due to the adsorption of mouse serum albumin in process of pre-incubation, but the particles were found in the hepatocytes. Interestingly, hollow mesoporous silica nanoparticle (HMSNs) loaded with perfluoro-15-crown-5-ether (PFCE) adsorbed more proteins than the bare particles, especially the amount of apolipoproteins A-1 and A-2. These proteins only induced ENPs to accumulate in liver 24 h after injection, but in other RES organs like spleen or lung (Pochert et al., 2017). In summary, the biodistribution of ENPs determines the organ/tissue specific therapeutic or toxic effects, and the PC formation plays an important role. Undesirable biodistribution of NPs in disease treatment applications will reduce their therapeutic effect and even induce toxicity. Therefore, controlling PC formation in nanomedicine can provide more precise guidelines for improving the specificity and efficiency of NPs for targeted biodistribution.

### Metabolism

Engineered nanoparticles structural change and eventual excretion in either original form or metabolites through different pathways is the process of degradation or metabolism, also known as biotransformations. PC is known to affect the biotransformation of ENPs *in vivo*. Until now, most of studies revealed that the PC impacts ENPs’ metabolic and degradation rate through affecting blood circulation and biodistribution. For example, the MRI traceable superparamagnetic mesoporous silicon nanoparticles (MaPSi) adsorbed proteins that were mainly associated with liver activity, immune response, coagulation, and wound response. Those were distinguished from that of dual PEGylation (DPEG) modified MaPSi (DPEG-MaPSi) (which were enriched with glycoproteins CD59, CD44, CD47, CD93, and CD36). Such difference caused DPEG-MaPSi to have significantly longer circulation half-life (241 min) than that of MaPSi (1 min) (Figure 1D; Nissinen et al., 2016). Moreover, the composition of PC on the surface of NPs@PEG was rich in albumin, which is different with that on NPs@Glc (enrich fibrinogen). It induced NPs@PEG core to have a faster degradation rate than NPs@Glc, in both liver and spleen (Stepien et al., 2018). Reducing protein binding affinity can also increase circulation half-life of ENPs. Compared with PLGA-PEG NPs (100% methoxy-PLGA20K-PEG5K), maleimide-functionalized PLGA-PEG NPs can increase circulation half-life from 3.48 to 22.2 h by reducing protein binding affinity (Zhou et al., 2018). Until now, the impact of PC on ENPs biotransformations is mainly focus on *in vitro* experiments. Many issues *in vivo* remain needed to be unraveled. Since PC is endogenous and dynamical, and is the outermost layer of NPs, it is involved in the early process of biodegradation of ENPs. Thus, their degradation products may affect the fate of ENPs *in vivo*. Furthermore, various types of protein compositions may have different effects on the biodegradation rate of ENPs *in vivo*. Therefore, optimization of the products and rate of ENPs’ biodegradation though controlling PC formation requires more attentions in future clinical application.

### Toxicity to Immune Systems and Others

**FIGURE 2:**
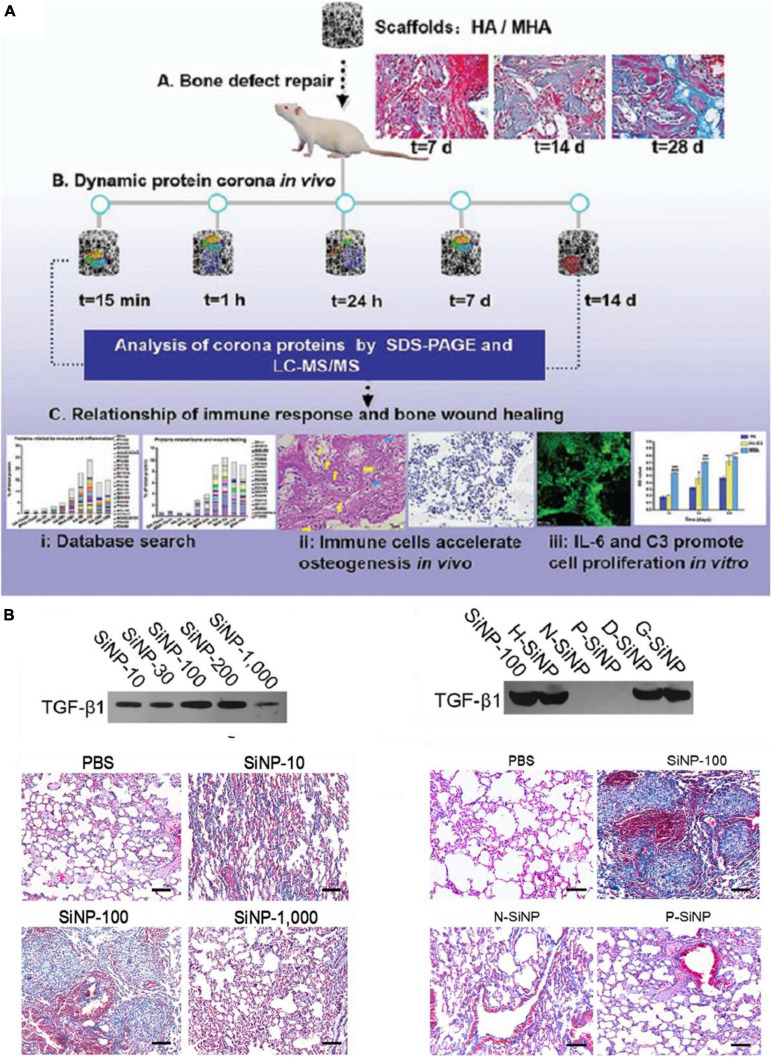
A few examples of PC-induced toxicity. **(A)** *In vivo* PC formation influenced immune-modulating osteogenesis by magnetic nanoparticle (MNP)-infiltrated bone regeneration scaffolds. Adapted with permission from Zhu et al. (2019). **(B)** SiNP-100 specifically recruited TGF-β1 to their corona and subsequently induced lung fibrosis (N-SiNPs, amination-SiNPs; P-SiNPs, polyetherimide-SiNPs). Adapted with permission from Wang et al. (2017).

The formation of PC impacts not only on the pharmacokinetics of ENPs but also ENPs’ toxicity including immunotoxicity. In general, PC formation changes the nanoparticle-cell interactions and may reduce cytotoxicity of ENPs through inhibiting their agglomeration and increasing the stability. The PC layer may also shelter ENP’s surface, thus reduce cytotoxicity caused by surface chemistry or metal ion release. Complement system is involved in the specific and non-specific immune mechanisms of the body (Merle et al., 2015a, b). As expected, complement proteins were shown to be involved in identifying and inducing the elimination process of ENPs. Thus, immunotoxicity is one of the main toxicity of ENPs induced by PC in body. For example, PEG-coated iron oxide NPs (IONP-PEG) can trigger complement activation and induces an inflammatory response (increased in proinflammatory cytokines such as IL-1β, IL-6, and TNF-α) (Rivera et al., 2019). Magnetic nanoparticle (MNP)-infiltrated bone regeneration scaffolds could also adsorb inflammatory related protein such as alpha-2-HS-glycoprotein, haptoglobin, and complement components, which can activate the immune system (Figure 2A; Zhu et al., 2019). In addition, Toy et al. (2019) found that imidazole-aceticacid (IAA) modification polyethyleneimine (bPEI) NPs adsorbed less fibrinogen than bPEI NPs, and thus have reduced immune activation and expression of chemokine expression. Moreover, blood hemolysis, liver toxicity, and anaphylactic responses were also reduced. However, for IAA-modified chitosan NPs, their toxicity was decreased mainly through the balance of TLR and complement activation.

Besides immunotoxicity, other types toxicity can also be regulated by PC *in vivo*. For example, one study explored SiNPs with a diameter of 100 nm and special chemistry such as hydration (H-SiNPs), dextran (D-SiNPs), and gelatin (G-SiNPs), can induce lung fibrosis by specifically recruit and enrich transforming growth factor β1 (TGF-β1) into their PC in lung (Figure 2B; Wang et al., 2017). Besides, SiNP-100 not only kept biological activities of TGF-β1 in binding cell receptors and triggering lung fibrosis, but also slowed degradation rate and prolonged activation of the TGF-β/Smad2 pathway which directly promotes lung fibrosis. In addition, amorphous silica NPs with a diameters of 70 nm (nSP70) induced acute lethality and abnormal activation of coagulation cascade, which attributed to the special affinity of silica NPs for coagulation factor XII in blood (Yoshida et al., 2015). In another case, the amino modified nSP70 showed lower toxicity compared to nSP70 due to fewer coagulation factor XII adsorbed on the surface. Therefore, toxic effects have emerged to be one of the main problems that need to be solved in nanomedicine (Dhawan et al., 2018). Since PC is the outermost surface that interacts with cells and tissues, it affects the toxicity of ENPs as well as their functions. On one hand, PC can decrease the toxicity of ENPs, which may improve their performance. On the other hand, PC may also shield their targeting capability and induce immunotoxicity. Therefore, precise understanding and taking the advantages of PC provide opportunities as well as challenges in the design of medical-used ENPs.

## Modulation and Applications of *in vivo* PC

### Modulation of *in vivo* PC

**FIGURE 3:**
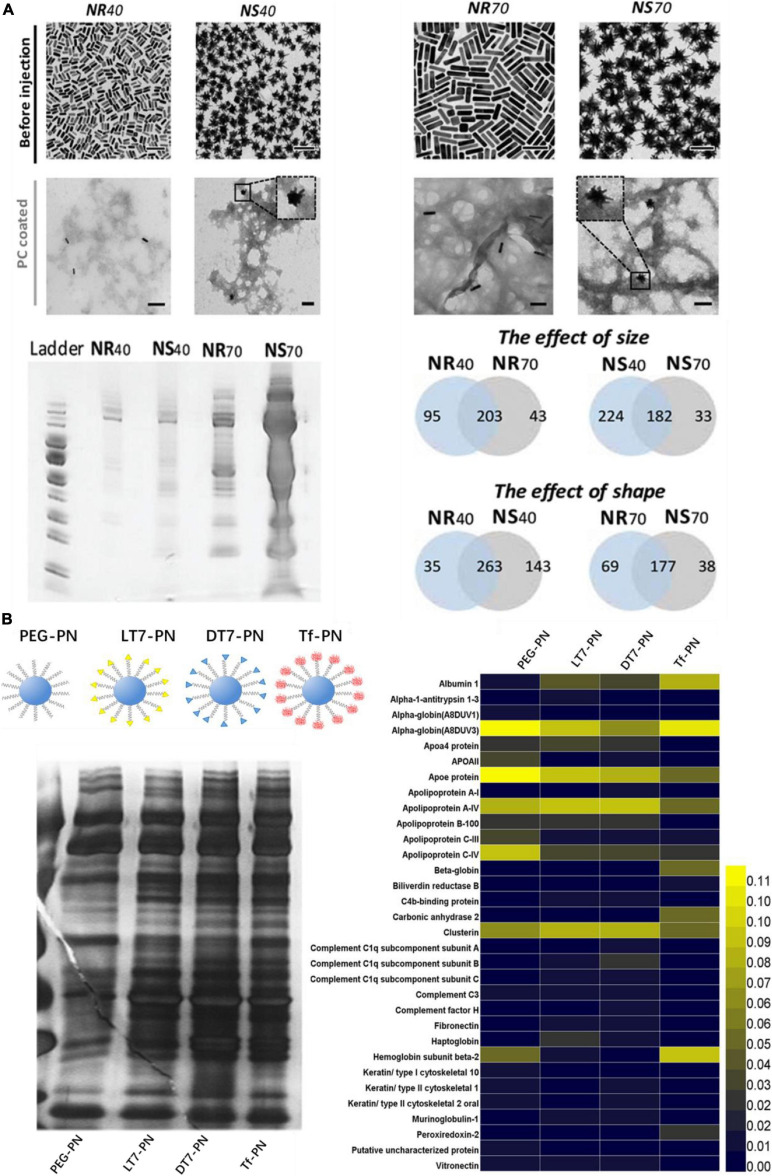
Physicochemical properties of ENPs affect the *in vivo* PC formation. **(A)** TEM images of AuNPs with different size and shape and they adsorbed protein amounts and types *in vivo*. Adapted with permission from García-Álvarez et al. (2018). **(B)** Four types of polystyrene NPs modified with PEG and Tf receptor (TfR)-targeting ligands (LT7, DT7, and Tf) and their corresponding *in vivo* PC formation. Adapted with permission from Zhang et al. (2018).

Since PC plays an essential role in regulating bio-nano interactions *in vivo*, modulation or control the PC formation is an urgent need to address. As a dynamic process, the PC formation is not only highly dependent on the properties of ENPs, but also on the biological environment. Physicochemical characteristics of ENPs such as size, shape, composition and surface chemistry modulate PC composition (Yu et al., 2020). ENPs size determines their surface curvature and also affects the surface area. Size is an important factor that affect not only the number but also the types of proteins adsorbed on the surface of ENPs *in vivo*. One study presented the effect of size (40 and 70 nm) on the formation of AuNSs *in vivo* (García-Álvarez et al., 2018). The amount of proteins adsorbed on AuNSs with a diameter of 70 nm were about 9 folds more than that of AuNSs with a diameter of 40 nm. On the contrary, protein types that adsorb on AuNSs with a diameter of 40 nm was about 88.8% higher than that of AuNSs with a diameter of 70 nm (Figure 3A). The similar effect of size on PC also presented on AuNRs with the same size and surface chemistry. Besides, due to surface curvature effect, unlike flat surfaces, proteins adsorb on the surface of ENPs alter their conformations to fit the curved surface, and this result in different protein binding affinities. It has been found that 35 nm AuNPs covered with artificial virus NPs (AVNs), which generated by integrating lipids, showed a much more prominent soft corona formation than that of 80 nm AuNPs with the same coating (Xu et al., 2016).

The morphology or shape has a great impact on the structure and composition of PC *in vivo*. One study showed that amount of protein adsorbed on AuNSs with a diameter of 40 and 70 nm were 2 and 9 folds more than that of AuNRs with the same size, respectively (Figure 3A). It may attribute to the higher surface area for NSs Furthermore, with a diameter of 40 nm, the number of protein adsorbed on the surface of AuNSs was about 36% more than that of AuNRs (García-Álvarez et al., 2018). On the contrary, the number of proteins adsorbed on the surface of AuNRs with a diameter of 70 nm was about 14% more than that of AuNSs with the same size.

Due to the various properties, surface chemistry is another crucial factor influencing the formation and evolution of PC on ENPs. Surface charge is an important factor for regulating PC *in vivo*. It has been found that the types of protein adsorbed on neutral SPIONs and negatively charged SPIONs were about 72 and 59% more than that of positively charged SPIONs *in vivo*, respectively (Sakulkhu et al., 2014). Apolipoprotein A-II was found in the PC of neutral and negatively charged SPIONs, but not in the PC of positively charged SPIONs. The PCs formed on the surface of positively charged NPs contained considerably higher amounts of low molecular weight (<30 kDa) proteins, which is different from neutral SPIONs (high amount of protein with molecular weight <30 kDa and 50∼70 kDa). In addition, ligand density can also regulate PC *in vivo*. Bertrand et al. (2017) synthesized PEG–PLGA NPs with different PEG densities, from10 to 50 PEG chains per 100 nm^2^, and they found that PEG–PLGA NPs with higher PEG densities appeared to have lower relative abundance of apolipoprotein E. Zhang et al. (2018) synthesized four types of polystyrene NPs modified with PEG and Tf receptor (TfR)-targeting ligands (LT7, DT7, and Tf), and they found that different amounts and types of proteins adsorbed on the NPs, in which PEG-modified polystyrene NPs have the lease PC adsorbed (Figure 3B). Moreover, complement proteins were significantly abundant in PC of DT7 modified NPs.

Besides the properties of ENPs, environmental factors are also important for regulating PC. Generally, temperature, pH, fluidics, and concentration of protein are the main environmental factors for regulating PC *in vitro*. However, *in vivo*, PC is strongly affected by personal features in blood, such as a patient’s specific disease. Therefore, different compositions of PC can also be found in different individuals, even incubated with the same NPs. This gives rise to a concept of personalized PC (PPC). Colapicchioni et al. investigated the composition of PC on AmBisome-like liposomes in breast, gastric, and pancreatic cancer patients (Caracciolo V. et al., 2016). They found liposomes adsorb more protein in pancreatic cancer patients than that in breast and gastric patients. Especially for proteins with molecular weight ∼37 kDa, which are associated with immunoglobulin alpha (IgA) and IgG.

The modulation of *in vivo* PC is a comprehensive task, as ENPs are complex systems by possessing parameters from several dimensions including size, shape, composition, surface chemistry, etc. Among these properties, surface chemistry is heavily investigated as the ENP’s biological properties are largely surface-driven. Even a single surface chemistry change can alter the surface charge, hydrophobicity, hydrogen bonding, π bonding, or topography structure of an ENP. Because a construct of ENP involves multiple aspects of parameters, it’s extremely challenging to obtain a thorough relationships between ENPs’ physiochemical properties and PC formation. Thus, some observations achieved from one type of ENP may not extend to another ENP without a complete control of these parameters. Besides, the complex biofluids including protein density, types, and structures can subtly differ from one to another in different studies. Furthermore, as these nanomedicine research in small animals are ultimately used in humans, it is also critical to understand the physiological differences between different species regarding the *in vivo* PC formation.

### Applications of *in vivo* PC

Adsorption of protein on the surface of ENPs and formation of PC-ENP complex *in vivo* seem inevitable. Thus, we can obtain specific biofunctionalized PC-ENP complex with unique advantages in biomedical applications. At present, the study of PC has gradually evolved from identification to application. There have been applied researches in the field of biomedicine such as drug carriers, targeting and personalized therapy based on PC.

Engineered nanoparticles used for targeted delivery of cancer drugs *in vivo* have been extensively investigated (Chou et al., 2011; Yu et al., 2012). There are two main targeting strategies for ENPs’ delivery to tumor: passive targeting and active targeting. However, most investigations remain at the laboratory stage. One possible reason is that PC rapidly formed on the surface of the carrier, thereby impeding their targeting (Salvati et al., 2013; Su et al., 2016, 2018). For passive targeting, ENPs target tumors by enhanced permeability and retention (EPR) effect. However, proteins such as immunoglobulins, fibrinogen, and complement can facilitate the clearance of ENPs by MPS. Thus, decrease of the adsorption of these types of protein on EPS can be used to enhance their passive targeting. For example, Takeuchi et al. (2017) prepared molecularly imprinted nanogels (MIP-NGs) with the capability of albumin recognition, which allows the particles to be immediately cloaked by albumin corona after injection, and thus reduces phagocytosis. As a result, most of MIP-NGs accumulated in tumor tissue, and almost no retention in liver tissue. Besides, PEG-conjugated lipid nanoparticle (LNP) adsorbed apolipoproteins in blood and successfully delivered small interfering RNA (siRNA) to LDLR-expressed HepG2 tumors and, with the accumulation ratio of tumor to all organs up by about 30% (Chen et al., 2019). Tesarova et al. (2020) found that PAS [proline (P), alanine (A), and serine (S)] modified ferritin (FRT)-based nanocarriers (PAS-10-FRTElli) reduced PC formation and did not activate complement C3 in mice. They were used to deliver cytostatic alkaloid ellipticine (Elli) *in vivo*. Compared with Elli alone or FRTElli, the amount in Elli internalization after treatment with PAS-10-FRTElli in tumor increased by nearly 3 folds. On the contrary, the amount in Elli internalization after treatment with PAS-10-FRTElli in liver decreased by 58% compared with that of non-encapsulated Elli. Compared to unmodified FRTElli, the amount in Elli internalization in spleen after treatment with PAS-10-FRTElli decreased by about 55%. Besides, DTX encapsulated in maleimide-modified ENPs were also used to target breast tumor site/cells (Li et al., 2018). Compared with unmodified ENPs, maleimide-modified ENPs adsorbed higher amount of low-immunogenic albumin, which protected ENPs from phagocytosis and prevented accelerated blood clearance. Then, maleimide-modified NPs carrier specially accumulated in tumor by albumin receptor-mediated active targeting or passive targeting in tumor tissue, and released DTX in tumor cell, followed by enhancing antitumor activity. Therefore, for drugs delivered by passive targeting, *in vivo* PC formation should be considered. Decreasing the composition of immunoglobulins, fibrinogen, and complement will prolong the blood circulation time and improve the targeting efficiency. Some surface modifications to modulate PC formation are needed in nanocarrier construction.

**FIGURE 4:**
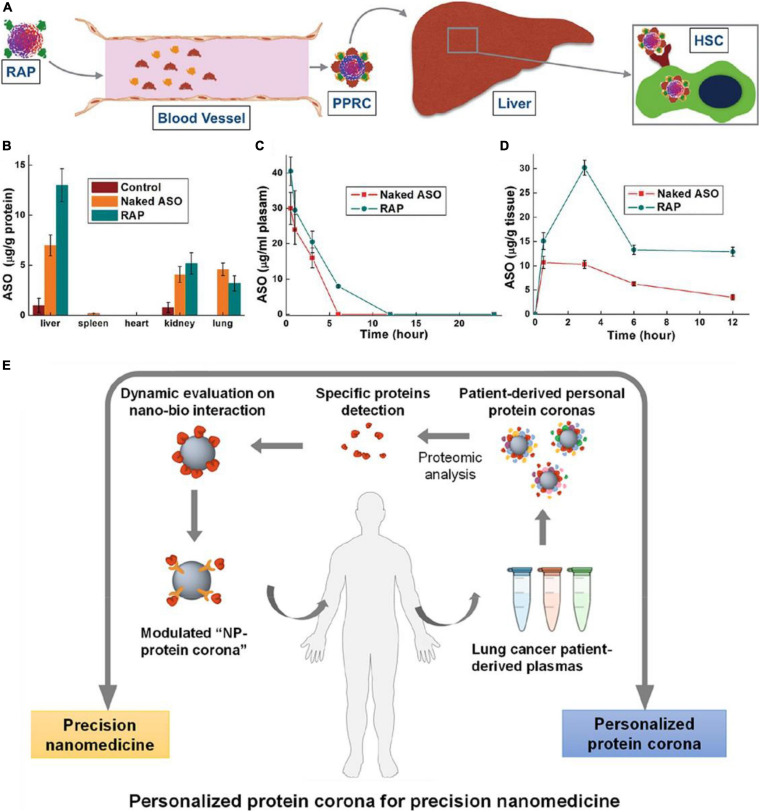
Sample applications of *in vivo* PC formation in nanomedicine. **(A)** Schematic diagram showing the fate of ASO-loaded retinol-conjugated polyetherimine (RAP) NPs after intravenous administered. **(B)** ASO (naked or delivered by RAP NPs) distribution in different organs. **(C,D)** Time-dependent ASO (naked or delivered by RAP NPs) concentrations in **(C)** the blood and **(D)** the liver. Adapted with permission from Zhang et al. (2015). **(E)** Schematic illustration of nanomedicine development strategy based on systematic analysis of patient-personalized PCs. Adapted with permission from Ren et al. (2019).

For active targeting, ENPs are modified with affinity ligands for specific recognition by the targeted cells via receptor mediated pathways. PC can also impact active targeting by their various compositions. For example, through controlling surface PC, Zhang et al. (2015) delivered antisense oligonucleotide (ASO) using the retinol-conjugated polyetheramine NPs system for the treatment of liver fibrosis (Figures 4A–D). This nanocarrier can selectively recruit retinoic alcohol binding protein 4 in the protein environment, which targets hepatic stellate cells (HSC). Then the ASO was released in HSC specifically and therefore effectively inhibits the expression of type I collagen, and thus reduces the liver fibrosis in mouse models. In addition, after modified with retinol, poly(beta-amino ester) polymers combined with terminal oligopeptides (OM-PBAE) increased the adsorption of apolipoproteins in the corona, thus enhanced active targeting to the liver, where receptors for these proteins are located (Fornaguera et al., 2019). 2-mercapto ethanol modified AuNSs can reduce the amount of adsorbed PC, and thus enhanced the active targeting ability of 2Rb17c nanobody to epidermal growth factor 2 receptor expressing tumor cells (D’Hollander et al., 2017).

Since PC formation can highly impact the adsorption, biodistribution, and activity of nanocarriers, engineered PC formation and controlled PC compositions have raised much attention and huge interests recently. One of most common strategies is surface chemistry modulation. The above research works using this approach successfully modulated PC formation and improved the passive or active targeting efficiency of nanocarriers. Besides, PC pre-coating before nanocarriers were administered has emerged as a novel and facile strategy to engineer PC formation *in vivo*. For example, pre-coating of ApoE reduced the adsorption of albumin and immunoglobulin E on graphene/gold NPs, which enhanced the blood circulation time and tumor targeting (Lu et al., 2019).

Personalized therapy based on *in vivo* PC formation is another application. As ENP-plasma protein interactions are strongly influenced by the composition of PC, such interactions are highly dependent on the source of plasma. Therefore, it is speculated that different NP-protein complex exist between individuals, especially patients with specific diseases. Thus, understanding of PPC not only benefits the nanotherapy treatment efficacy but also aid in disease diagnosis. For example, Ren et al. (2019) found that Gd@C_82_(OH)_22_ NPs adsorbed more C1q in lung cancer patient than that in healthy human. In lung cancer patient, binding affinity between C1q and Gd@C_82_(OH)_22_ is significantly high, and secondary protein structure of C1q was abnormal, which subsequently influences the biological functions of C1q such as immune response. Thus, C1q can be as a specific biomarker for cancer diagnosis and cancer immune therapy (Figure 4E). Furthermore, the amount and composition of PC adsorbed on poly(ethylene glycol) (PEG)-coated mesoporous silica with a diameter of 100 nm (PEG-MS-100) were different from plasma samples of 23 healthy donors (Ju et al., 2020). Such changes of PC on PEGylated doxorubicin-encapsulated liposomes, especially for immunoglobulins (IGKV2-29, IGHV4-34, IGHG1) and complement proteins (C4B, C3), can impact particle-immune cell interactions. Thus, based on the diversity of plasm proteins in patients with different disease or in healthy person, a new class of size- and shape-tunable personalized protein NPs (PNP) was designed and made from patient-derived proteins (Lazarovits et al., 2019). The PNPs are advantageous because they are biodegradable, biocompatible, modifiable and non-toxic *in vivo*. They can be further combined with unique molecular fingerprints from different human patients, providing a huge potential in personalized nanomedicine.

## Conclusion and Perspectives

After administered or exposed to living organisms, ENPs’ biobehaviors are highly related to their activity and safety. Besides a synthetic identity, PC endows ENPs a new biological identity, and subsequently alters the biobehaviors of ENPs. PC-related biological effects are dependent on their compositions and structures. Therefore, PC characterizations are critical. However, obtaining the accurate PC information remains a challenge, due to rapid and dynamic exchanges of proteins, limit of sample processing time, and weak interactions in some cases. Accurate characterization of *in vivo* PC formation is more difficult compared to *in vitro* characterization due to their complex environment. Until now, pioneer works on this aspect were focused on PC formation in blood. As compositions of PC significantly change over time in different cells, tissues, and organs, the spatial-temporal specific PC information still cannot be obtained. As the blood PC formation is dynamic, the findings of relationships between PC and biological effects of ENPs with static approaches may not be accurate. Moreover, PC compositions were identified from a pool of ENPs, which reveal the average level of protein adsorption. In fact, unlike small molecules, each individual ENP may vary slightly on physiochemical properties and the *in vivo* PC formation on each ENP is likely to be different, which poses another challenge of understanding the mechanisms.

*In vivo* PC formation is known to determine the *in vivo* behaviors of ENPs including absorption, blood circulation, biodistribution, metabolism, and toxicity, which are important pharmacokinetic parameters for ENPs used for nanomedicine development. So far, only a few studies have obtained the relationships between PC compositions and *in vivo* behaviors to predict the ENPs’ biological effects. Furthermore, PC is not only one layer of adsorbed proteins but can be two or more layers. The outmost layer of proteins directly influences the ENPs’ biobehaviors. Therefore, mostly reported relationship studies obtained from analysis of the total PC might not be accurate. Future research should pay attention to distinguish the inner adsorbed proteins and outer adsorbed proteins.

Engineered nanoparticles can be functionalized by specific targeting moieties to target disease sites in nanomedicine, however, the *in vivo* PC formation can completely or partially shield the targeting ability of ENPs. Therefore, modulating *in vivo* PC formation to reduce the negative influences of PC is important. *In vivo* PC formation can be modulated by the size, morphology, charge, and surface chemistry of ENPs. Besides, recruiting specific proteins to realize targeting delivery of ENPs has become a new concept. Additionally, *in vivo* PC formation can also be affected by different types of blood as the plasma proteome is different between individuals. In this case, a systematic investigation of disease-related *in vivo* PC formation is beneficial for diagnosis and therapeutics, as well as speeding up the clinical translation of ENPs. In the future, we can envision disease-specific and *in vivo* PC-based diagnostic and therapeutic ENP agents.

## Author Contributions

XB, QM, and GS designed this work of review. XB and JW performed the literature search of the databases. XB and GS wrote the manuscript. QM revised the manuscript. All authors have read and approved to publish the final manuscript.

## Conflict of Interest

The authors declare that the research was conducted in the absence of any commercial or financial relationships that could be construed as a potential conflict of interest.
